# Few-photon microwave fields for superconducting transmon-based qudit control

**DOI:** 10.3762/bjnano.16.112

**Published:** 2025-09-11

**Authors:** Irina A Solovykh, Andrey V Pashchenko, Natalya A Maleeva, Nikolay V Klenov, Olga V Tikhonova, Igor I Soloviev

**Affiliations:** 1 Lomonosov Moscow State University, Faculty of Physics, Moscow, 119991, Russia; 2 Lomonosov Moscow State University, Skobeltsyn Institute of Nuclear Physics, Moscow, 119991, Russia; 3 All-Russian Research Institute of Automatics n.a. N.L. Dukhov (VNIIA), 127055, Moscow, Russia; 4 Moscow Technical University of Communications and Informatics (MTUCI), 111024, Moscow, Russia; 5 National University of Science and Technology ”MISIS”, 119049, Moscow, Russia; 6 Kotel’nikov Institute of Radio Engineering and Electronics of RAS, 125009 Moscow, Russia

**Keywords:** Josephson “atoms”, non-classical fields, quantum state control, superconducting qubits

## Abstract

Increasing the efficiency of quantum processors is possible by moving from two-level qubits to elements with a larger computational base. An example would be a transmon-based superconducting atom, but the new basic elements require new approaches to control. To solve the control problem, we propose the use of nonclassical fields in which the number of photons is comparable to the number of levels in the computational basis. Using theoretical analysis, we have shown that (i) our approach makes it possible to efficiently populate on demand even relatively high energy levels of the qudit starting from the ground state; (ii) by changing the difference between the characteristic frequencies of the superconducting atom and a single field mode, we can choose which level to populate; and (iii) even the highest levels can be effectively populated on a sub-nanosecond time scale. We also propose the quantum circuit design of a real superconducting system in which the predicted rapid control of the transmon-based qudit can be demonstrated.

## Introduction

Currently, quantum computing is under active development, opening new horizons for solving a number of problems that are difficult for classical processors, including modeling the behavior of quantum systems, optimization problems, breaking cryptographic systems, solving large systems of linear equations, and analyzing heat conduction equations [1–6].

The basis for the physical implementation of these computations is a quantum processor consisting of computational cells called qudits, whose states can be represented with satisfactory accuracy in the form of a decomposition into *n* basis states. Today, the main focus is on processors based on qubits (a special case of qudits with *n* = 2) on a superconducting, ionic, or other platform. However, it is still not easy to create the necessary number of qubits and control channels to implement really useful quantum algorithms. A promising solution to this problem is to expand the computational basis of an element by switching to qutrits (*n* = 3), ququarts (*n* = 4), and so on [7–12].

We believe that an additional synergistic effect can be achieved by using quantum electromagnetic fields with a comparable (with *n*) number of photons to control such quantum multilevel systems. The coexistence of different photons in a single waveguide should make it possible to use the scarce control circuits on a quantum chip more efficiently. In the future, the analysis of the behavior of “qudits + multiphoton quantum field” systems will form the basis for the practical implementation of quantum internet and telecommunication systems [13–16].

Among the many possibilities, we will focus on a superconducting platform; it allows one to create sources and mixers for microwave photons, qubits, and qudits with corresponding characteristic frequencies of transitions between basis states, as well as radiation detectors with the claim of being quantum-sensitive [17–26].

So far, the most common artificial atom among the superconducting ones is considered to be a charge qubit with a large shunt capacity, namely a transmon [27–29]. The transmon is technically simple to fabricate, easy to operate, and resistant to decoherence from various sources. Transmon-based qudits are already being used to detect microwave fields [30]. The latter feature makes it possible to achieve a long lifetime of this artificial atom; in a recent work [29] “coherence” times of *T*_1_ = 64–13 μs, Ramsey periods of *T*_2R_ = 85–16 μs, and Hahn echo times *T*_2E_ = 93–22 μs for levels of *n* = 2–10, respectively, have been achieved.

It should be noticed that the spectrum of eigenvalues of the Hamiltonian of a real transmon (a slightly nonlinear oscillator) is quite close to the equidistant one; however, a number of widely used theoretical models describing its evolution in an external electromagnetic field (the Jaynes–Cummings model) do not take into account the high-lying energy levels of the artificial atom, nor the nonlinearity existing in a real solid-state system [31–33]. In our fully quantum analysis of the “atom”–field interaction, the nonlinearity of transmon will be taken into account.

This article presents the results of a theoretical description of the interaction between a few-photon microwave non-classical field and a transmon-based qudit with several, even high-lying, levels being taken into account. We develop methods of rapid quantum control of the designed transmon-based qudit and its state population dynamics. The structure of the article is as follows: First, the model of the system under study is described in more detail, followed by a theoretical description of the Fock-based control of the qudit states and a discussion of possible practical implementations.

## Results

### Model description

**Figure 1:**
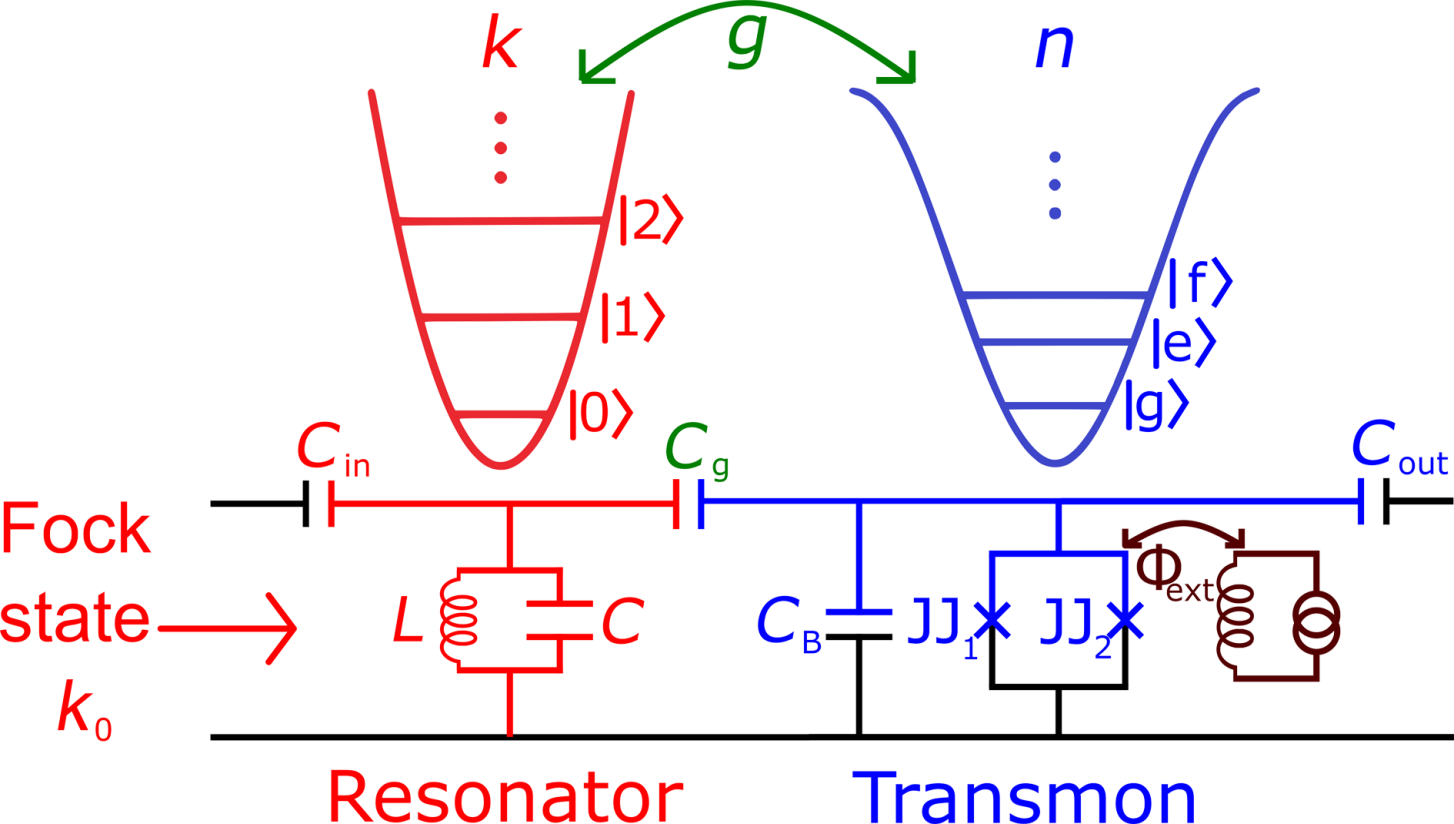
Schematic representation of the model under discussion: A few-photon microwave field from a high-quality resonator (red) affects an artificial transmon-based atom (blue). The potential energies and energy spectra for harmonic (resonator) and anharmonic (transmon) oscillators are shown above. Crosses mark the Josephson junctions in the transmon interferometer. The external magnetic flux Φ_ext_ is used to tune the spectrum of a transmon.

The system under consideration consists of a high-quality superconducting resonator (the quality factor is about 10^5^–10^6^ and depends mainly on the external coupling *C*_in/out_) connected to a transmon [34] by a capacitance *C*_g_ (see Figure 1). The resonator in this system is a quantum harmonic oscillator with a fully equidistant energy spectrum described by the bosonic ladder operators 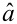 and 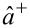, and the photon number operator 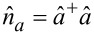. The transmon is considered as an anharmonic oscillator (with ladder operators 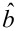 and 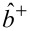) with the number of excitations in the solid-state system similarly introduced as 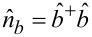. In a transmon, the inductance is created using a nonlinear element, that is, a nanoscale Josephson junction (JJ), or a pair of JJs forming an interferometer-like circuit, so the spectrum is no longer equidistant. In the case where the JJ pair is used, the characteristic (plasma) frequency of the transmon can be quickly adjusted in 10–20 ns in the range of 1 GHz by an external magnetic field [35]. In practice, researchers try to reduce the transmon frequency dependence on the external magnetic field to get rid of parasitic flux fluctuations. A large shunt capacitance *C*_B_ is needed to increase resistance to parasitic charge fluctuations [36].

A few-photon non-classical microwave field (with a certain number of photons, *k*_0_) enters the resonator [37–42] with variable frequency detuning Δω between the resonator and the artificial atom. The time evolution of the quantum state of the transmon qudit, the populations of its eigenstates, and the number *n* of excitations induced in the superconducting system by the quantum field is studied. By taking into account the nonlinearities in the system, it will be shown that there is a certain value of the frequency detuning at which the dynamics of the energy transition from the field to the solid-state system and vice versa is most efficient.

### Theoretical description of Fock-based qudit control

First, we need to quantize the field in the harmonic oscillator that corresponds to a high-quality resonator. The energy of the electric field stored in the capacitor and the energy of the magnetic field stored in the inductor can be written as follows:


[1]

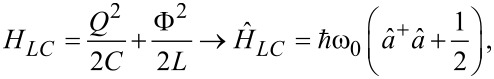



with operators of quantum charge and flux introduced by:


[2] $$\hat{Q}=iQ_{\text{zpf}}\left({\hat{a}}^{+}-\hat{a}\right),\;\hat{\Phi }=\Phi_{\text{zpf}}\left({\hat{a}}^{+}+\hat{a}\right),$$

where


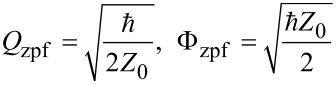


are the vacuum fluctuations of charge and flux, *Z*_0_ is the characteristic impedance, and 
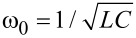
 is the resonator angular frequency. The transmon is treated almost the same way, but, in this case, the number of Cooper pairs on the shunt capacitor *C*_B_ (island) and the phase on the JJ/(interferometer) are quantized as follows:


[3] $${\hat{n}}_{\text{CP}}=\frac{i}{2}{\left(\frac{E_{\text{J}}}{2E_{\text{C}}}\right)}^{\frac{1}{4}}\left({\hat{b}}^{+}-\hat{b}\right),\hat{\varphi }={\left(\frac{2E_{\text{C}}}{E_{\text{J}}}\right)}^{\frac{1}{4}}\left({\hat{b}}^{+}+\hat{b}\right),$$

where charge energy *E*_C_ = e^2^/2*C*_B_ and Josephson energy *E*_J_ = (Φ_0_*I*_c_)/2π are used (*I*_c_ is the critical current flowing through the Josephson junction). The Hamiltonian for the transmon part of our system can be written in the following form, taking into account the nonlinearity [6]:


[4]

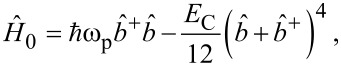



where -*E*_C_/12 is the nonlinearity parameter and 
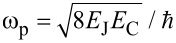
 is the plasma frequency of the transmon.

The first term in the Hamiltonian describes a free linear evolution of the photon operators, characterized by their oscillations in time in the Heisenberg representation. The nonlinear term in the Hamiltonian can be averaged over high-frequency oscillations, leaving only smoothly varying terms. This procedure actually corresponds to the so-called rotating wave approximation, in which the following type of nonlinear term can be obtained:


[5] $${\left(\hat{b}+{\hat{b}}^{+}\right)}^{4}\approx 6{\hat{n}}_{b}^{2}+6{\hat{n}}_{b}+3.$$

The expression for the nonlinear term obtained in Equation 5 indicates that the nonlinearity of the transmon is similar to the type of Kerr phase modulation 
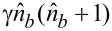
, with 
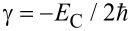
. Thus, the Hamiltonian in Equation 4 can be rewritten as follows:


[6]

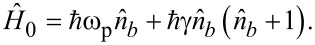



Note that, for such a system, the operator 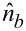 is found to be independent on time (being an integral of motion). This means that this nonlinearity itself leads only to phase modulation without changing the excitation statistics.

In our case, the dynamics of the excitations of a Josephson nanosystem (transmon) under the action of a nonclassical electromagnetic field is studied. The interaction of the photonic and superconducting subsystems is investigated by direct solution of the nonstationary Schrödinger equation:


[7]

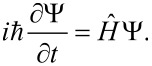



The Hamiltonian of such a system, taking into account both the nonlinearity of the transmon and the transmon–field coupling, can be written as follows:


[8]

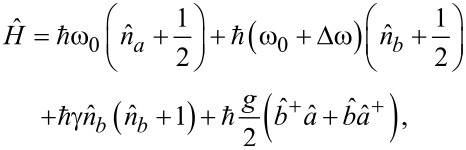



where ω_0_ + Δω = ω_p_ is the transmon frequency. The interaction strength of the resonator mode with the Josephson subsystem is taken as 
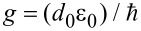
, where 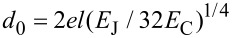 is the dipole moment of the transmon,


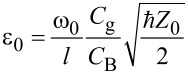


is the vacuum electric field in the resonator that affects the transmon, and *l* is the distance that the Cooper pair travels when tunneling through JJ [43]. The conditions for the application of the rotating wave approximation, which makes it possible to use the relation in Equation 5, are Δω ≪ ω_0_ and *g* ≪ ω_0_ [44].

Here, the efficiency of the interaction between two subsystems is determined by the average photon density ⟨N⟩/*V*_res_, which is large enough to allow field-induced transitions to occur significantly faster than any decoherence processes in the system [29]. This actually corresponds to the strong-field regime and makes it possible to correctly describe the dynamics of a quantum system in terms of the nonstationary Schrödinger equation without taking dissipations into account [45].

The developed theoretical approach appears to be very powerful and allows one to describe the mutual influence between the superconducting and field subsystems beyond the perturbation regime with efficient excitation of transmon being taken into account. For the case of few photons in the field mode, the analytical solution of the problem is found. In the general case, the nonstationary Schrödinger equation (Equation 7) was solved numerically using the expansion of the total non-stationary wave function in terms of the interaction-free eigenfunctions of the Josephson, ϕ*_n_*, and field, 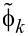, subsystems:


[9]

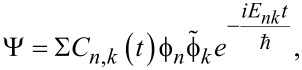



where the designation of the total energy in the system is


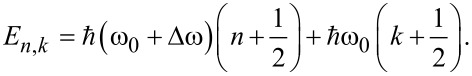


Substituting the solution in Equation 9 into Equation 7 leads to a system of differential equations for probability amplitudes *C_n_*_,_*_k_*(*t*) to find *k* photons in the field mode and *n*-fold excitation of the transmon:


[10]

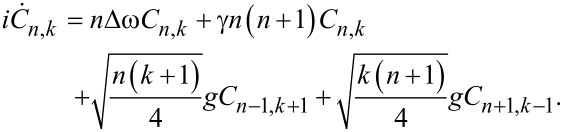



Based on the obtained solution, the probability of detecting a transmon in the state with the number *n* is given by:


[11] $$P_{n}\left(t\right)=\Sigma_{k}{\left|C_{n,k}\left(t\right)\right|}^{2}.$$

The probability of finding *k* photons in the field mode can be found similarly to Equation 11 as follows:


[12] $$W_{k}\left(t\right)=\Sigma_{n}{\left|C_{n,k}\left(t\right)\right|}^{2}.$$

The initial state is considered to be the Fock state of the resonator with the number of photons *k*_0_ denoted as 
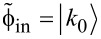
.

## Discussion

### Different regimes of transmon population dynamics

**Figure 2:**
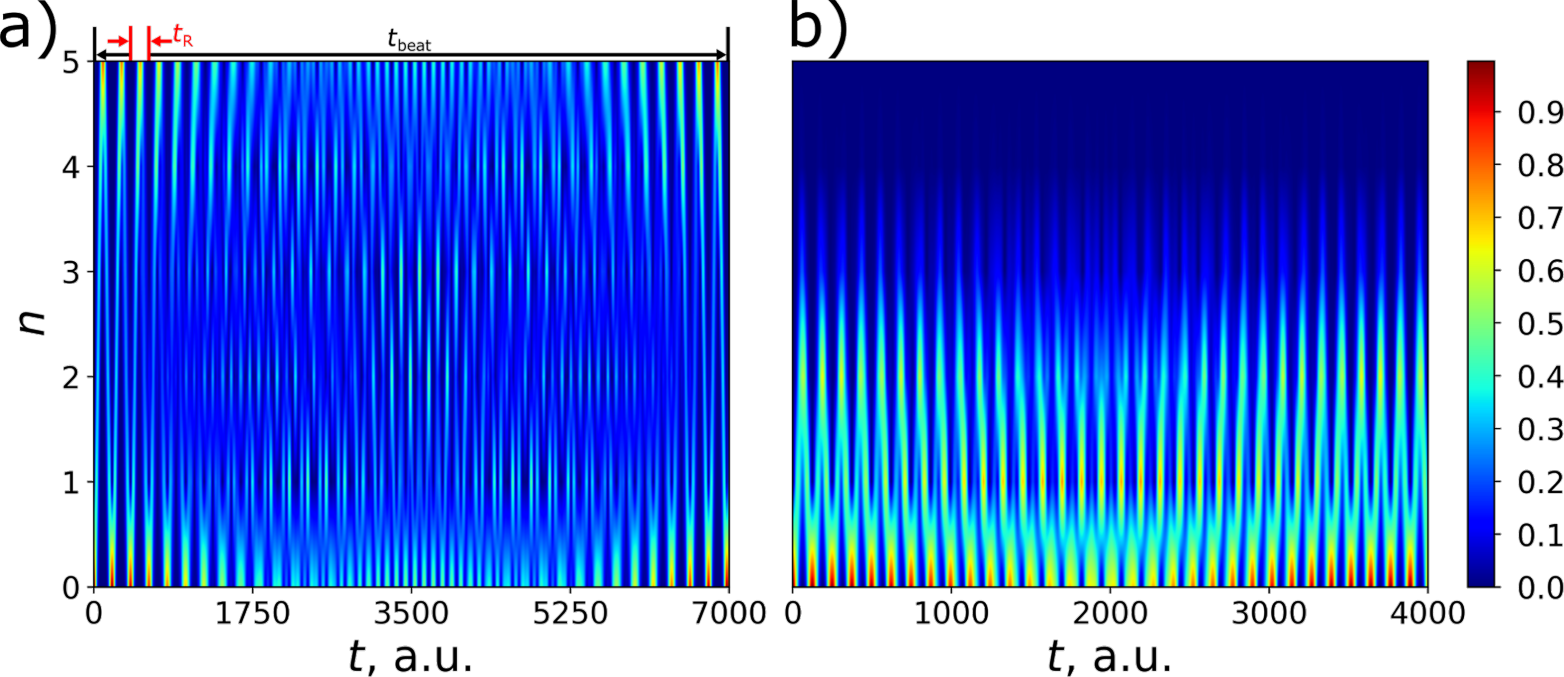
Distribution (colored) of the probability of transmon state excitation versus time with initial state 
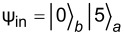
 for nonlinearity parameters (a) γ = −0.001ω_0_ and (b) γ = −0.01ω_0_ for *g* = 0.03ω_0_, Δω = 0. The dimensionless time unit is *t*, which is converted to dimensional units via the formula τ = 2π*t*/ω_0_.

The first feature demonstrated for the interacting superconducting subsystem and a single-mode quantum field is a significant influence of the Josephson nonlinearity (which is similar to the Kerr self-phase modulation) on the dynamics of the transmon excitation. Figure 2 shows 2D distributions characterizing the time dynamics of the population of different transmon states in the case of strong and weak nonlinearity in the system. Here we see Rabi-like oscillations [46–48] between different transmon states, and the amplitude of these oscillations is characterized by slow modulation resulting from the nonlinearity effect. It is shown that even a small nonlinearity leads to the appearance of amplitude modulation, and different numbers of states are characterized by different modulation and frequency. Moreover, it is found that significantly different regimes of dynamics take place in dependence on the value of the key parameter *K*, which combines the characteristics of both the nonlinearity and coupling with the quantum field:


[13] $$K=\frac{\gamma k_{0}\left(k_{0}+1\right)}{g}.$$

Actually, this parameter represents the ratio between the efficient nonlinearity of the transmon and the strength of its coupling with the quantum field. It is very important that the efficient nonlinearity is calculated for maximal possible transmon excitation directly determined by the initial number of photons in the field *k*_0_. For relatively small values of the nonlinearity parameter (*K* ≪ 1), a strong coupling between the field and the Josephson subsystem gives rise to periodic transition of the transmon to high-energy states, as can be clearly seen in Figure 2a. Here, all the energy initially stored in the field can be transferred to the transmon with periodic maximal population of the highest possible excited transmon state with *n* = *k*_0_.

An increase in the nonlinearity of the transmons leads to a significant reduction in the period of Rabi oscillations, *t*_R_, and the beat frequency, *t*_beat_, as shown in Figure 2a. When nonlinear interactions dominate, high-energy excitations are strongly suppressed. This fact is illustrated in Figure 2b.

### Population control through frequency detuning

As it was shown in the previous section, the regime of strong nonlinearity, when the parameter *K >* 1, leads to suppression of excitation of high-energy transmon states. However, here we propose and discuss a method how to overcome this effect. We have found out that it is possible to controllably manage the excitations in the Kerr nonlinear transmon by varying the frequency detuning of Δω. Using the law of energy conservation in the case of the initial state 
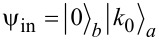
, we have analytically found the formula to determine the optimal value of the frequency detuning that produces the maximum excitation of a certain transmon state “on demand”:


[14]

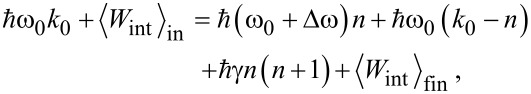



where ⟨*W*_int_⟩_in/fin_ denotes the average value of the interaction energy in the initial and final states of the system, respectively. For an exact number of exitations in the system, the average interaction energy is zero, which means that for the case of the initial state of the transmon, ⟨*W*_int_⟩_in_ = 0. In addition, under the condition of ensuring the maximum possible excitation, no energy should be involved in the interaction in the final state, so ⟨*W*_int_⟩_fin_ = 0. Thus, Equation 14 implies an expression for the optimal frequency detuning at which the maximum excitation of the state with the highest number *n* = *k*_0_ can be achieved:


[15] $$\Delta \omega_{{\text{opt}}_{n}}=-\gamma \left(n+1\right).$$

**Figure 3:**
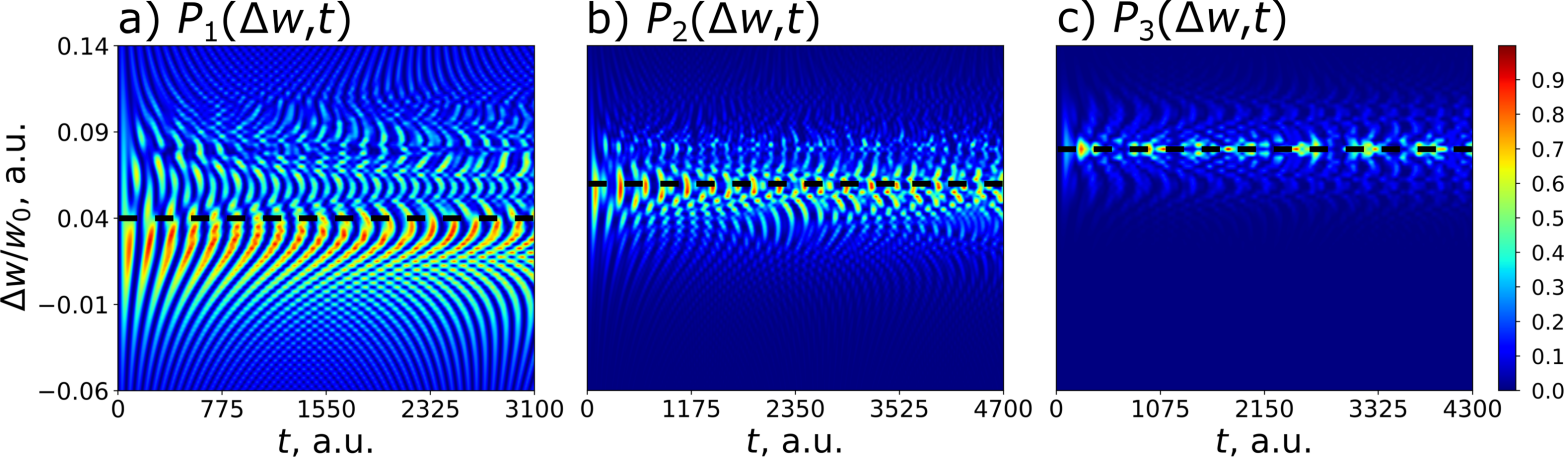
Distribution (colored) of the probability of transmon state excitation versus time of (a) the first, (b) the second, and (c) the third of the transmon states at *g* = 0.025ω_0_ and γ = −0.02ω_0_ as a function of the frequency detuning Δω for the initial state 
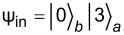
. The horizontal black dashed lines correspond to the theoretical values of the detuning 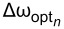 obtained from Equation 15. As the transmon levels *n* increase, the theoretical detuning match the maximum detunings from numerical simulations Δω*_n_*.

It should also be emphasized that this analytical method, based on finding the integral of motion, makes it possible to predict the optimal frequency detuning without solving the system of Equation 10. Equation 15 is explicitly confirmed by the numerically calculated 2D probability distribution of the excitation of different transmon states shown in Figure 3 in dependence on frequency detuning and time.

A very well-pronounced maximum of the probability is found at optimal frequency detuning at each of the three presented distributions. It is important to note that Equation 15 is valid and can also be applied in the case of any intermediate transmon state, but in this case the characteristic peak width for the level population can be large enough to lead to some overlapping and interference patterns in the distribution (see Figure 3a,b). Physically, these lateral peaks occur in other settings when not only the desired state is involved in the excitation, but also some other neighbors. In this case, the average interaction energy in Equation 14 becomes non-zero, providing a different energy state that leads to additional preferred values of the frequency detuning.

**Figure 4:**
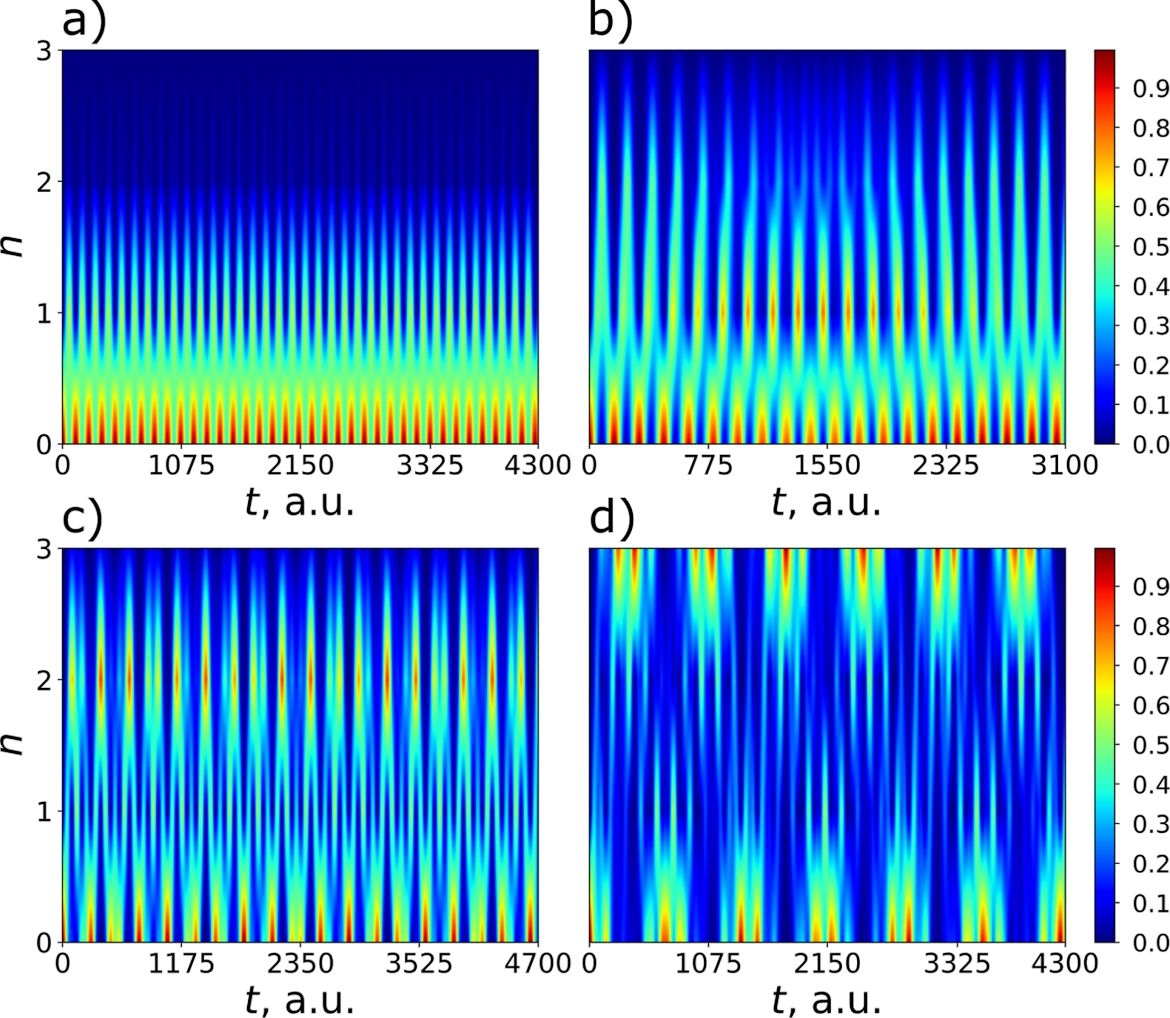
Distribution (colored) of the probability of transmon state excitation versus time, obtained in the case of the initial state 
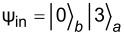
 in the regime with predominant nonlinear interaction (*K* = 9.6) for *g* = 0.025ω_0_ and γ = −0.02ω_0_. Panel (a) corresponds to the resonant case at Δω = 0, panel (b) corresponds to the optimal frequency for efficient population of the first state Δω = 0.04ω_0_, panel (c) corresponds to the optimal frequency for efficient population of the second state Δω = 0.06ω_0_, and panel (d) corresponds to the optimal frequency for efficient population of the third state Δω = 0.08ω_0_.

To demonstrate more precisely the possibility of highly efficient excitation of any transmon state “on demand” by frequency detuning, we calculate the time-dependent populations of transmon levels at optimal points. The results are shown in Figure 4. In the resonance case (Figure 4a), the excitation of high-energy transmon states is strongly suppressed due to significant influence of the Kerr nonlinearity (*K* = 9.6). However, it is clearly seen that the frequency adjustments found by Equation 15 for the first (Figure 4b), second (Figure 4c), and third (Figure 4d) Fock states are indeed optimal values, providing increased excitation of the considered states. The effect of possible maximum excitation is especially pronounced for the highest transmon level when all the input energy of the quantum field is transferred to the superconducting subsystem. Thus, the optimal frequency detuning allows one to overcome the suppression of excitation induced by strong nonlinearity and to achieve a periodically maximum population of a certain transmon state “on demand”.

### Quantum circuit design

The optimal frequency detuning opens the possibility to achieve maximum excitation of a certain transmon state even under strong nonlinearity. In practice, however, the case where *K* is close to unity may be strongly demanded. This regime corresponds to a rather strong coupling between the transmon and the quantum field and can be attractive due to the possibility of much faster transmon dynamics. Moreover, as will be discussed below, the experimental control of the excitation is much easier in this case. This regime is difficult to achieve in traditional qubit-based experiments, where everyone deals with the weak coupling regime when *g*/2π ≈ 10 MHz and γ/2π ≈ −100 MHz. Devoret et al. [49] showed that the coupling of the JJ system with the resonator can be significantly enhanced by placing it in the gap of the central conductor of the coplanar waveguide. In this case, the JJ system will interact directly with the current (magnetic field) in the cavity, and the coupling strength will change from 
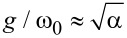
 to 
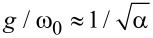
, where α is a fine structure constant. This case corresponds to the so-called “ultrastrong coupling regime” [50–51], which is beyond the scope of this article.

Later, it was shown that this system is inconvenient for practical implementation because the low nonlinearity of *E*_C_/*h* ≈ 5 MHz and the huge intrinsic capacitance of JJ *C*_J_ ≈ 4 pF are difficult to achieve. The reason was that the JJ system was located in the center of the resonator and inductive coupling prevailed. The problem can be solved by using the so-called “in-line transmon” design, that is, one should move the JJ system closer to the edge of the resonator in the area of the maximum voltage in the standing wave, where capacitive coupling will be implemented. At the same time, the value of the coupling strength will decrease, but will still remain quite large in comparison to the characteristic nonlinearity, that is, *E*_C_/*h* ≈ 300 MHz [52–53].

**Figure 5:**
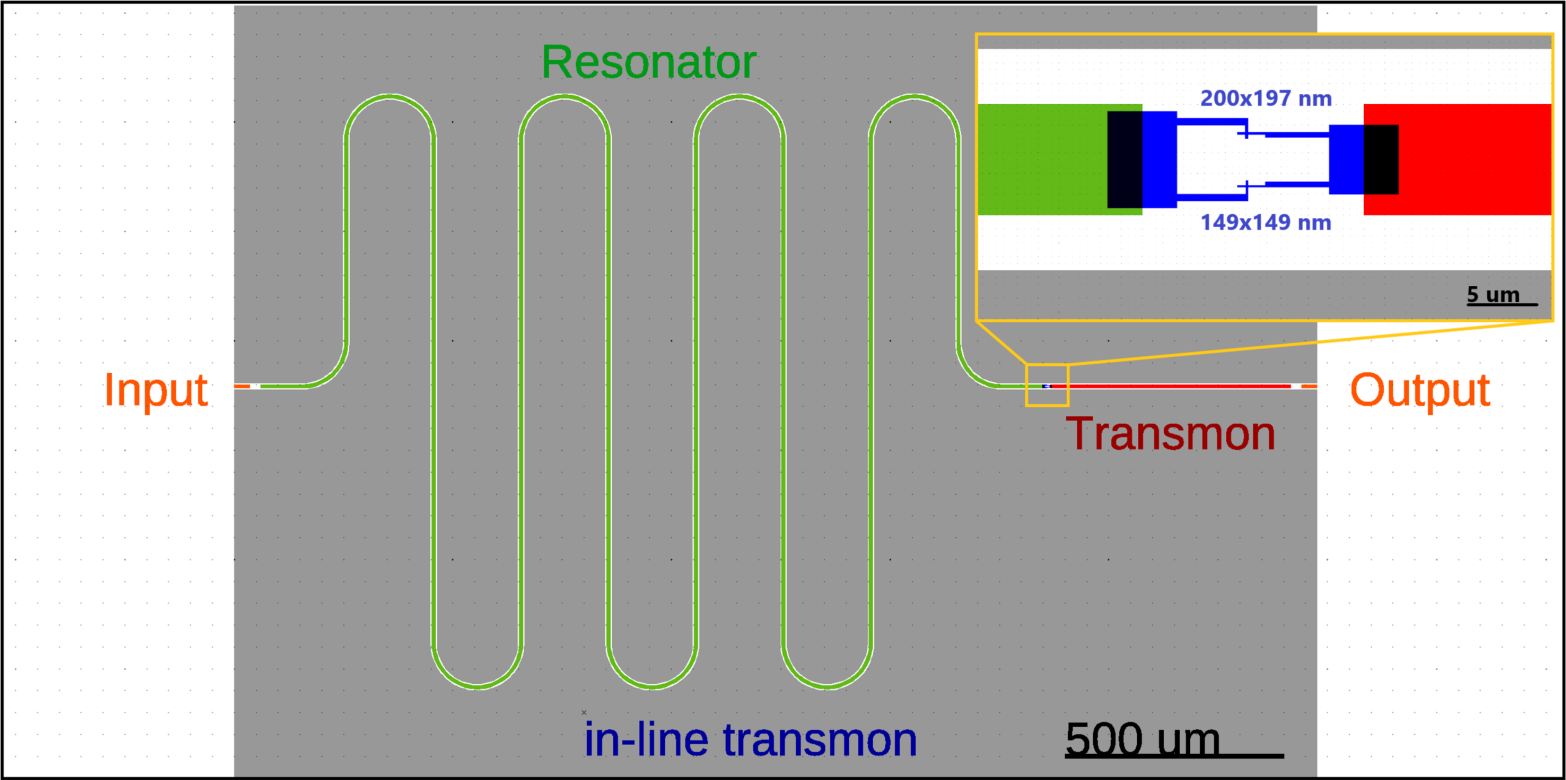
In-line transmon design for efficient transmission of the quantum state. The red part of the central conductor of the resonator and the blue SQUID form a transmon, the total length of the resonator (green and red parts) forms the main resonant mode ω_0_.

In this article, we turned this concept into a realistic design to demonstrate the experimental feasibility of the proposed qudit control with microwave photons. In our case, the characteristic magnitude of the nonlinearity γ/2π = −*E*_C_/2*h* = −100 MHz is directly proportional to the charge energy *E*_C_ of the transmon, which is determined by the capacitance of the remaining part of the resonator *l_q_* = 549 μm (Figure 5, the red part of the resonator). The coupling strength can be estimated as:


[16] $$\frac{g}{2\pi }=\sqrt{\frac{2\pi Z_{0}\alpha }{Z_{\text{vac}}}}{\left(\frac{E_{\text{J}}}{2E_{\text{C}}}\right)}^{\frac{1}{4}}\frac{\omega_{\text{p}}}{2\pi },$$

and it will vary depending on the external magnetic flux (*E*_J_(Φ_ext_) and ω_p_/2π(Φ_ext_)). Here, *Z*_vac_ ≈ 377 Ω and *Z*_0_ = 50 Ω. Taking this expression into account, at a typical plasma transmon frequency of ω_p_/2π = 5–6 GHz, the coupling strength will be *g*/2π ≈ 1.2 GHz, and efficient state control of the qudit will be possible for low-energy levels, *n* = 1–3.

**Figure 6:**
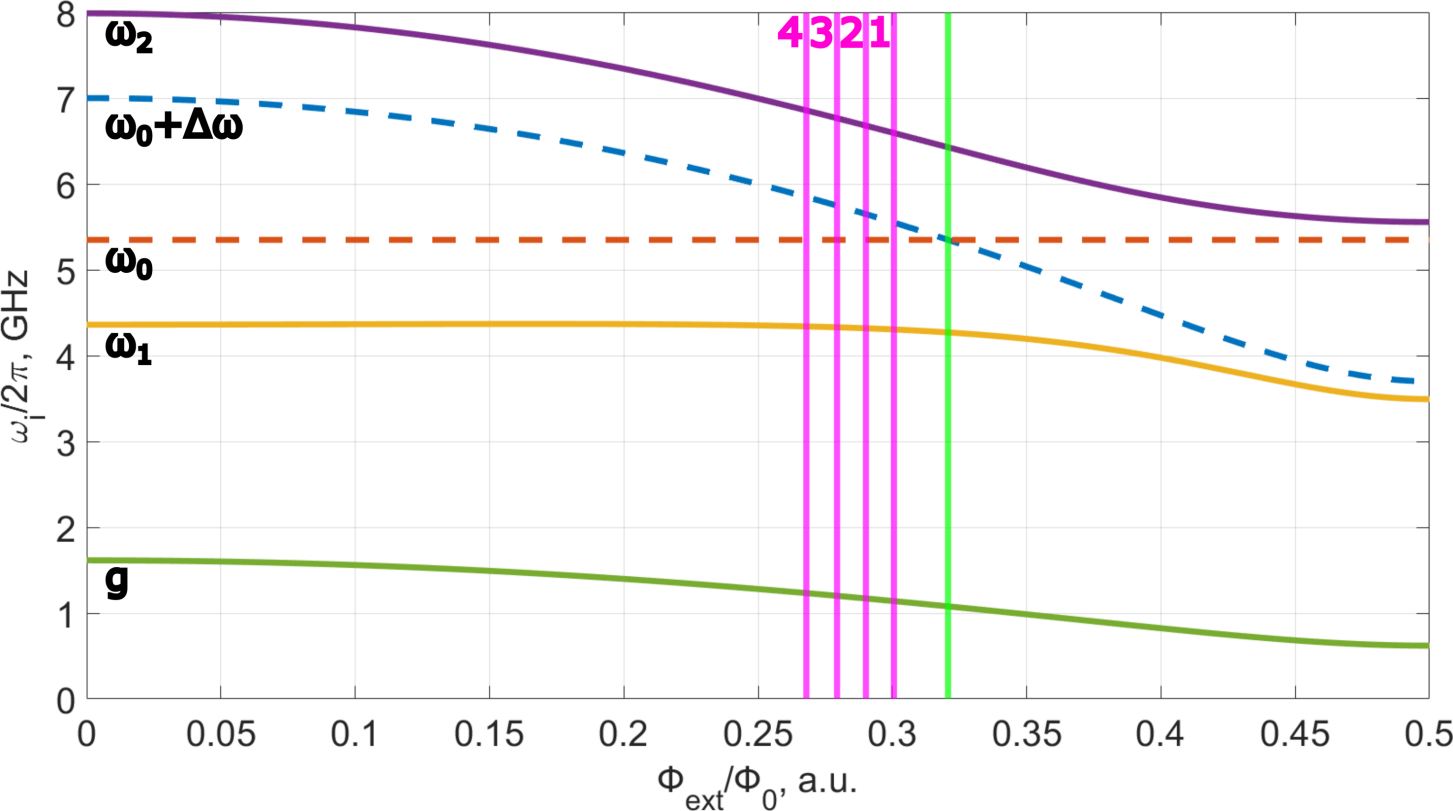
The dependence of the plasma frequency of the qudit ω_p_ = ω_0_ + Δω and coupling strength *g* between this two systems on the external magnetic flux Φ_ext_. When these two systems are connected, the united system with two modes ω_1_ and ω_2_ appears. Anticrossing at the point when the frequencies of the two systems coincide corresponds to the green vertical line.

Switching between effectively populated states is carried out when an external magnetic flux Φ_ext_ is applied to the interferometer, taking into account the condition 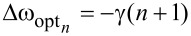 (Figure 6). The tuning of the plasma frequency is regulated by the interferometric arm asymmetry, and the values of *E*_J_ determine the magnitude of the critical current and the area of each JJ: *I*_c1_ ≈ 39.44 nA, *S*_1_ = 200 × 197 nm^2^, *I*_c2_ ≈ 22.21 nA, and *S*_2_ = 149 × 149 nm^2^, with the usual critical current density of *j* = 1 μA·μm^−2^. The frequency of the resonator was chosen to be ω_0_/2π = 5.348 GHz to provide simultaneously strong coupling with the quantum field and optimal detuning from the resonance. In addition, this frequency determines the total length of the system, that is, 2*l* = 11.101 mm.

Let us discuss the limitations on the values of the physical parameters in this scheme. First of all, the following relation between Josephson and charge energies should be satisfied: *E*_J_ ≫ *E*_C_, provided in our design by the ratio *E*_J_/*E*_C_ ≈ 100, which correlates well with the chosen type of superconducting artificial atom. The second constraint *C_J_* ≪ *C_s_* = *l_q_C*^0^ ≪ 2*lC*^0^ is also satisfied (the capacity of JJ can be estimated as *C_J_* = εε_0_*S*/*d*, ε = 10, and *d* = 2 nm for an AlO*_x_* film).

**Figure 7:**
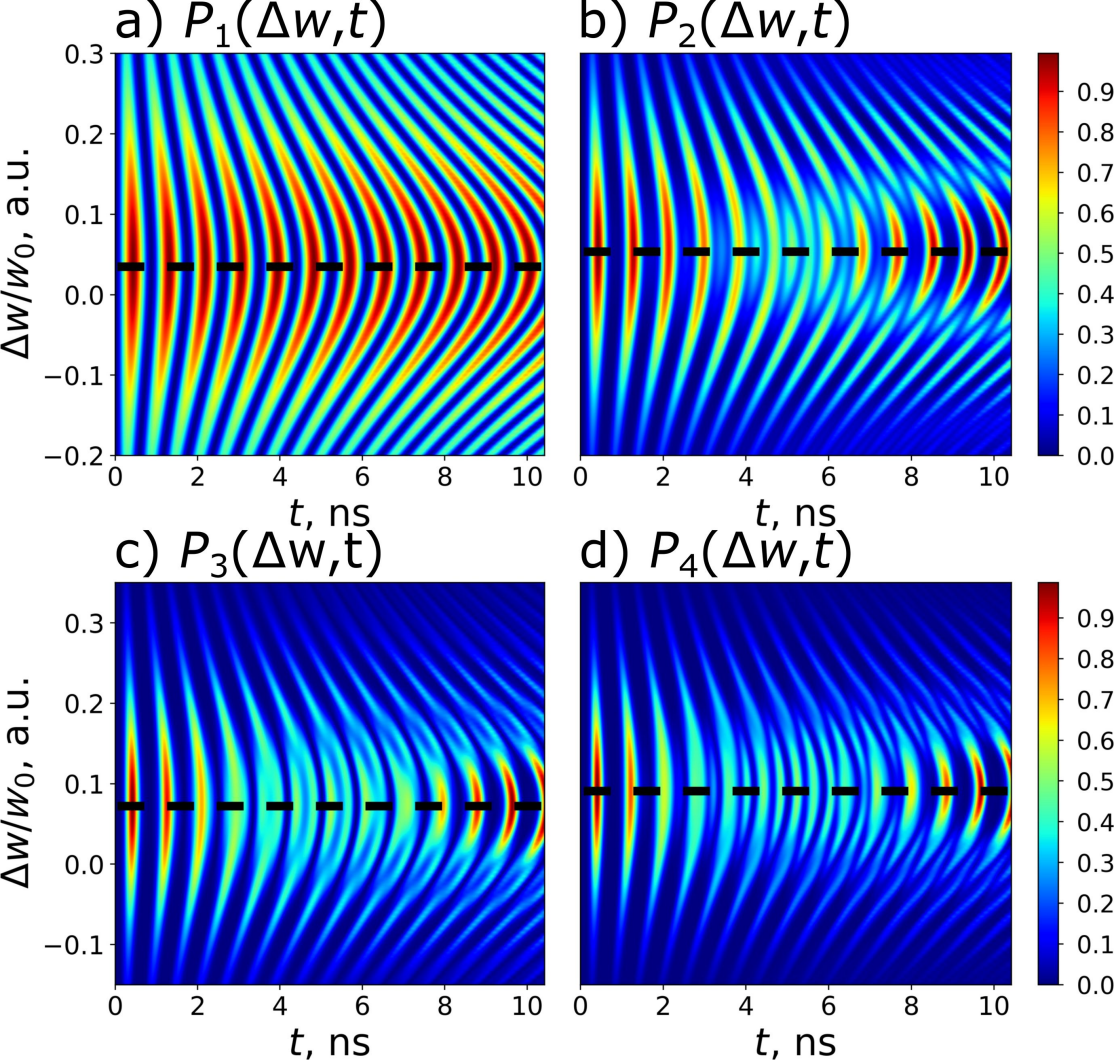
2D distributions of the populations of states with *n* = 1–4 of the designed transmon with nonlinearity γ = −0.0187ω_0_ calculated in dependence on frequency detuning and time at certain values of the coupling strength specific for each considered state: (a) *g* = 0.213ω_0_, (b) *g* = 0.219ω_0_, (c) *g* = 0.225ω_0_, and (d) *g* = 0.231ω_0_. *k*_0_ = 4 photons are chosen to be initially in the quantum field mode. The horizontal black dashed lines correspond to the theoretical values of the detuning (Equation 15), which is close enough to the numerical calculation results, see Table 1 for details.

This implementation has a number of significant drawbacks; the system takes up a lot of space on the chip, and the impedance matching for the JJ system and the resonator is a problem. Nevertheless, for this discussed in-line transmon design, all necessary parameters are calculated and values of the coupling strength *g* corresponding to the optimal transmon frequencies predicted by Equation 15 and providing the most efficient excitation are found for the four lowest transmon states. For each considered transmon state, its population is numerically calculated as a function of frequency detuning and time at the found coupling strength to confirm the designed optimal frequency condition. The results are shown in Figure 7 and Table 1; they obviously prove that the optimal detuning providing maximum excitation of each state explicitly coincides with the value obtained in the designed scheme according to Equation 15 by varying the external magnetic flux and represented by four pink vertical lines in Figure 6 with corresponding numbers.

**Table 1:** Comparison of theoretical detuning 
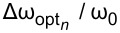
 with detuning Δω*_n_* obtained from numerical simulations.

*n*	1	2	3	4

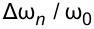 , a.u.	0.0374	0.0561	0.0748	0.0935
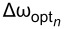 , a.u.	0.035	0.055	0.075	0.095

**Figure 8:**
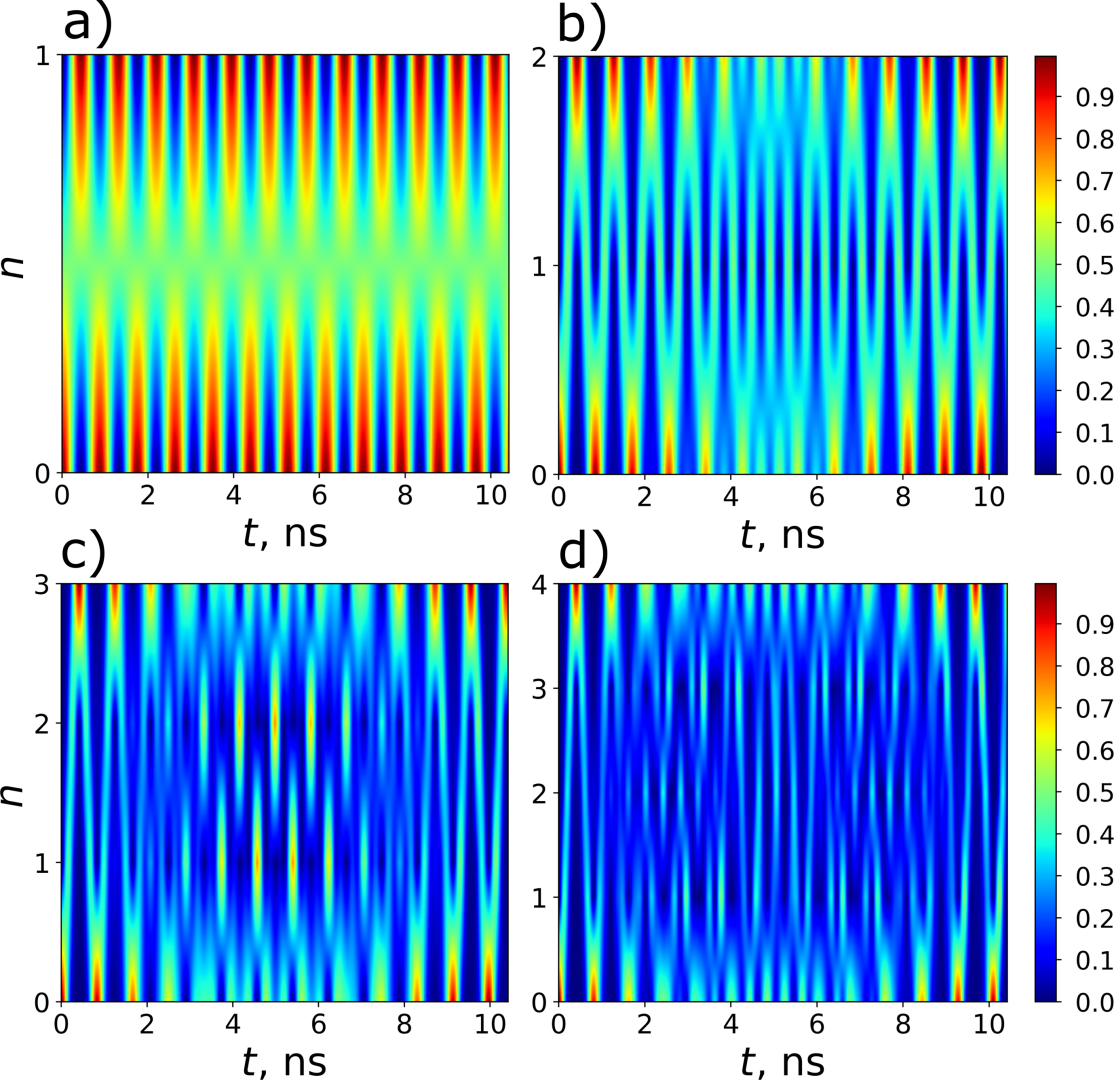
A demonstration of the rapid control of the states with *n* = 1–4 of the designed transmon (from (a) to (d), respectively). The panels show the time-dependent population of transmon states at the selected optimal frequency detuning. The parameters for each panel are the same as for the corresponding panels in Figure 7.

Moreover, it can be easily seen that the control of states is very rapid and can be performed on the sub-nanosecond time scale. Indeed, Figure 8 demonstrates the time-dependent probability of excitation of considered transmon states calculated for each state at its own optimal detuning. The obtained results demonstrate a very fast excitation with probability equal to unity achieved for each state of the designed transmon-based qudit, even for the highest one (Table 2). Thus, the strong-coupling regime appears to be very advantageous for the rapid sub-nanosecond control of the designed transmon-based qudit. In this case a very fine tuning to the optimal frequency can be performed by varying the applied magnetic flux.

**Table 2:** The maximum probability of excitation *P_n_* of the states with *n* = 1–4 of the designed transmon during the time τ*_n_*.

*n*	1	2	3	4

*P_n_*, a.u.	0.999976	0.995518	0.987228	0.975867
τ*_n_*, ns	0.438	0.427	0.417	0.405

## Conclusion

In this work, a fast, simple, and precise control of the population of an artificial atom is implemented theoretically using microwave photons (Fock states of the resonator). It is important to emphasize that, by adjusting the frequency of the nonlinear oscillator (qudit) from the linear resonator mode, we can choose which level of the solid-state subsystem is efficiently populated. Due to the nonlinearity, an efficient excitation of highly excited transmon states seems to be a challenging problem. It may be possible to excite this system “step by step” by choosing the appropriate frequency of the classical field for each stage, as was done in [54]. However, this procedure requires a rather complex experimental setup and takes a significant amount of time. For our particular system, we have identified and demonstrated the possibility of exciting a specific desired transmon state very quickly from the ground state (see Table 2 for details). We can significantly enhance the excitation of this specific transmon state by selecting an appropriate frequency detuning, whose value is determined analytically for each desired state. This phenomenon arises due to the interaction between nonlinearity and the coupling of quantum fields and cannot be observed when only classical fields are present.

In addition, we propose the quantum circuit design of a real superconducting scheme in which the predicted rapid control of transmon-based qudit can be demonstrated. It is important that, in a strong coupling regime, the efficient transitions in the transmon-based qudit occur on sub-nanosecond timescales [55–56]. Note that such times are not large in comparison to the decoherence process in the transmon-based qudit [29]. This circumstance makes it possible to design complex fully quantum hybrid “field + solid state” systems for quantum computing and developing a fully quantum interface between superconducting and photon platforms. Also, the developed transmon-based qudit can be used as an electromagnetic field detector, which allows one at least to determine the exact number of photons in the resonator.

## Data Availability

All data that supports the findings of this study is available in the published article and/or the supporting information of this article.
